# Radiological Factors Associated with Bisphosphonate Treatment Failure and Their Impact on Fracture Healing in Postmenopausal Women with Osteoporotic Vertebral Fractures

**DOI:** 10.3390/jcm12113820

**Published:** 2023-06-02

**Authors:** Hong Jin Kim, Ha Kyun Chang, Dong-Gune Chang, JiYun Ha, Byeong-Rak Keum, Gun-Hwa Kim

**Affiliations:** 1 Department of Orthopedic Surgery, Gyeonggibukbu Regional Military Manpower Administration, Seoul 11642, Republic of Korea; hongjin0925@naver.com; 2 Department of Obstetrics and Gynecology, Korea University Ansan Hospital, College of Medicine, Korea University, Seoul 15355, Republic of Korea; coolblue23@naver.com; 3 Department of Orthopedic Surgery, Inje University Sanggye Paik Hospital, College of Medicine, Inje University, Seoul 01757, Republic of Korea; dalang78@naver.com; 4 Department of Life Sciences, Pohang University of Science of Technology, Pohang 37673, Republic of Korea; br0104@kbsi.re.kr; 5 Research Center for Bioconvergence Analysis, Korea Basic Science Institute, Cheongju 34133, Republic of Korea; genekgh@kbsi.re.kr

**Keywords:** osteoporosis, osteoporotic vertebral fracture, bisphosphonate, bisphosphonate treatment failure, fracture healing, bone mineral density

## Abstract

(1) Background: Bisphosphonate treatment failure is one of the most difficult clinical problems for patients with osteoporosis. This study aimed to analyze the incidence of bisphosphonate treatment failure, associated radiological factors, and effect of fracture healing in postmenopausal women with osteoporotic vertebral fractures (OVFs). (2) Methods: A total of 300 postmenopausal patients with OVFs who were prescribed bisphosphonate were retrospectively analyzed and divided into two groups according to the treatment response: response (*n* = 116) and non-response (*n* = 184) groups. The radiological factors and the morphological patterns of OVFs were included in this study. (3) Results: The initial BMD values of the spine and femur in the non-response group were significantly lower than those in the response group (all Ps < 0.001). The initial BMD value of the spine (odd ratio = 1.962) and the fracture risk assessment tool (FRAX) hip (odd ratio = 1.32) showed statistical significance in logistic regression analysis, respectively (all Ps < 0.001). (4) Conclusions: The bisphosphonate non-responder group showed a greater decrease in BMD over time than the responder group. The initial BMD value of the spine and the FRAX hip could be considered radiological factors influencing bisphosphonate non-response in the postmenopausal patients with OVFs. The failure of bisphosphonate treatment for osteoporosis has a possible negative on the fracture healing process in OVFs.

## 1. Introduction

With the progressive increase in the elderly population, osteoporosis and osteoporotic vertebral fractures (OVFs) are important skeletal-related health problems that lead to a severe socioeconomic burden [1,2]. The development in anti-osteoporotic medications has had a substantial effect on bone mineral density (BMD) and the prevention of risk of fractures in various randomized studies [3,4,5,6]; however, there are some reports that have documented the negative effect of anti-osteoporotic medications on the physiologic bone remodeling and the fracture healing process [7,8].

Bisphosphonate as an anti-resorptive agent is the most common drug for osteoporosis but triggers some side effects, including gastrointestinal troubles, atypical femur fractures, and osteonecrosis of the jaw [7,8,9,10]. Thus, treatment of osteoporosis using bisphosphonate is important in the consideration of the resting phase [8]. Although there have been adequate responses for anti-osteoporotic medications from vast randomized controlled studies, some patients with osteoporosis do not have a substantial effect for anti-resorptive agents, with reported incidence rates ranging from 9.5% to 53% [11,12,13,14,15,16]. The causes of an inadequate response to bisphosphonate vary from poor compliance to secondary osteoporosis and hypovitaminosis D, thus masking the true incidence rate. On the basis of these causes, the International Osteoporotic Foundation has published guide-lines for addressing bisphosphonate treatment failure [17,18].

With the treatment failure of anti-osteoporotic medication, the healing potential of vertebral fracture from anti-osteoporotic agents must be considered in the patients with osteoporotic vertebral fractures [19,20]. For bisphosphonate, there was currently insufficient evidence as to whether it inhibits healing potential of fractures [20]. However, some radiographical indicators including IVC sign for non-union and morphological factors for instabilities provide a guidance for determining the fracture healing process [16,17,18,19,20]. Although the current guidelines suggest that anabolic agents provide a good efficacy of bone mineral density gains as well as the fracture healing effect, bisphosphonate is still used for the merits of the low costs and the ease of application in clinical practice [19,20,21]. Therefore, understanding the bisphosphonate treatment failure and radiological healing effect of vertebral fractures is important to treat postmenopausal women with OVFs.

Although the need for studies of treatment failure has been suggested before, studies for bisphosphonate treatment failure that exclude the known conditions capable of masking the true incidence are rare [11]. Given the existing lack of knowledge regarding bisphosphonate tolerance, the current choices for anti-osteoporotic medication have not been well studied from the evaluation of possible risk factors and the effect of fracture healing. Therefore, the aim of our study was to evaluate the incidence rates of bisphosphonate treatment failure, associated factors, and effect of fracture healing in postmenopausal women with OVFs.

## 2. Materials and Methods

**Figure 1:**
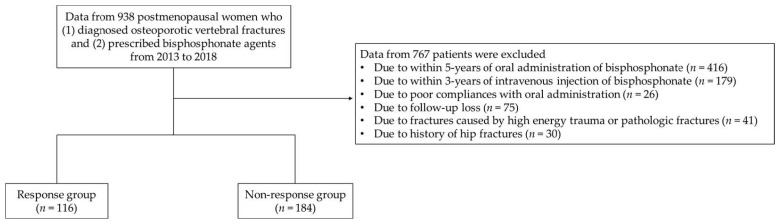
A flowchart of this study.

This study was conducted as a retrospective analysis of clinical and radiological data from patients at a single institute. Approval from the Institutional Review Board (IRB) was obtained, and the need for informed consent was waived by the IRB due to the retrospective design. A total of 983 postmenopausal women diagnosed with OVF and who were prescribed bisphosphonate agents after their diagnosis were included from 2013 to 2018. Exclusion criteria were those who within five years of oral administration or three years of intravenous injection showed the occurrence of fractures caused by high energy trauma and pathologic fractures; poor compliance with oral administration; and/or follow-up loss. Patients with a history of hip fracture and those with incidental hip fractures were also excluded to control other fracture-related variables in this cohort. All selected patients had no history of receiving estrogen therapy within 5 years. A total of 300 patients were finally included in this study and allocated into a response group (*n* = 116) or a non-response group (*n* = 184) according to the following criteria: (1) new incident fragility fracture or (2) a reduction in BMD that was greater than the least significant change (LSC) in the femur and/or spine with a basis of three years of intravenous agents (zoledronate) and five years of oral administration agents (alendronate), concerning the drug holiday period of bisphosphonate medication per the guidelines of the International Osteoporosis Foundation (Figure 1) [17]. The severity of osteoporosis was defined along the guidelines of the Endocrine Society Guideline Update [19,20].

All data were collected by an orthopedic surgeon using the hospital database and retrospectively analyzed. In all cases, after five years of follow-up for the oral administration of bisphosphonate or three years of follow-up for intravenous injection of bisphosphonate; patient age; duration after menopause onset; body mass index (BMI); route of administration; social history, including current smoking (≥5 cigarettes/day and/or ≥10 packs/year) [22], alcohol intake (≥1 time/week, drinking more than six units in one day) [23]; steroid medication; medical history, including hypertension, coronary artery occlusive disease, thyroid diseases, asthma/chronic obstructive pulmonary disease, chronic kidney disease, dementia, cerebrovascular accident, rheumatic diseases, and psychiatric diseases; BMD; and fracture risk assessment tool (FRAX) were assessed. A dual energy X-ray absorptiometry (DXA) scan was obtained to measure the BMD at the lumbar spine, total femur, and femur neck. The BMD was used to measure the T-score at the initial and last follow-ups, which are based on the guideline suggested by The International Society for Clinical Densitometry (ISCD). The reference data of T-score are based on the data from Korea National Health and Nutrition Examination Study (KNHANES). For bisphosphonate use, all patients took 1250 mg of calcium carbonate and 800 IU of cholecalciferol every day.

**Figure 2:**
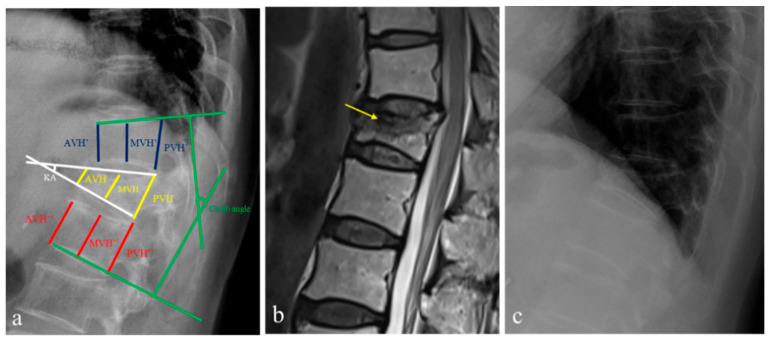
A 60-year-old woman who was diagnosed with an osteoporotic vertebral fracture (OVF) at T12. (**a**) To investigate morphological parameters, the kyphotic angle (KA), Cobb angle, anterior vertical height (AVH), middle vertical height (MVH), and posterior vertebral height (PVH) along with the AVH, MVH, and PVH of the adjacent upper and lower levels (AVH’ and AVH’’, MVH’ and MVH’’, PVH’ and PVH’’) were measured on lateral radiographs. From these parameters, the vertebral body compression ratio (VBCR), the percentage of anterior height compression ratio (PAHC), and the percentage of middle height compression ratio (PMHC) were calculated. (**b**) Sagittal magnetic resonance imaging showed an endplate type of T12 OVF with the presence of an intravertebral cleft sign (yellow arrow). (**c**) After two-years of follow-up with zoledronic acid medication, the VBCR, PAHC, and PMHC in lateral radiographs were also measured for the assessment of morphological factors.

The definition and morphological measurement methods for OVFs have not been well established yet. Thus, we compared reliable measurements and analysis to evaluate the fracture healing effect of OVFs [24,25,26,27]. From among the previously reported methods, the degrees of vertebral body collapse were measured on the basis of the vertebral body compression ratio (VBCR), the percentage of anterior height compression ratio (PAHC), and the percentage of middle height compression ratio (PMHC). These parameters included measurements from the anterior vertebral height (AVH), middle vertebral height (MVH), and posterior vertebral height (PVH), together with the AVH, MVH, and PVH of the adjacent upper and lower levels (AVH’ and AVH″, MVH’ and MVH″, PVH’ and PVH″) at the initial and 2-year follow-ups [28]. To analyze the morphological parameters for OVF, the following six observation markers were used: (1) VBCR (calculated using the AVH to PVH radio); (2) PAHC (calculated using the ratio of the AVH to the mean of the adjacent upper and lower levels); (3) PMHC (calculated using the ratio of the MVH to the mean of the adjacent upper and lower levels); (4) kyphotic angle (KA) (defined as the angle between the lower and upper borders of the fractured vertebrae); (5) Cobb angle (defined as the angle formed between a line drawn parallel to the superior endplate of one vertebra above the fracture and a line drawn parallel to the inferior endplate of the vertebra one level below the fracture) (6) the presence of an intravertebral cleft (IVC) sign (defined by a crescent-shaped shadow in the vertebral body) sign; and (7) magnetic resonance imaging (MRI) classification (stratified as endplate type and mid-portion type) (Figure 2) [29,30].

Statistical analysis was performed using the SPSS Statistics for Windows, version 21.0 (IBM Corp., Armonk, NY, USA). Normal distribution was confirmed using the Kolmogorov–Smirnov test. Regarding continuous variables, the Student *t*-test and Mann–Whitney test were used for parametric data and non-parametric data, as appropriate. Regarding categorical variables, the chi-square test and Fisher-exact test were used for parametric and non-parametric data, as appropriate. Linear regression was performed to determine the correlation between factors and the failure of the bisphosphonate treatment following OVFs (categorical dependent variable). Factors with *p* < 0.25 in the univariate analysis were considered to be significant variables, and a multivariate logistic regression analysis was performed to elucidate the association of these factors [27]. Regarding the BMD data in the regression analysis, statistical comparisons of groups required converting negative numbers to positive numbers to express odds ratios. To predict the risk factors selected by multivariate logistic regression data, receiver operating characteristic (ROC) curve analysis was performed, and the cut-off value was set at the maximum value of the Youden’s index. Statistical significance was set at *p* < 0.05.

## 3. Results

### 3.1. Incidence Rate of Bisphosphonate Treatment Failure

**Figure 3:**
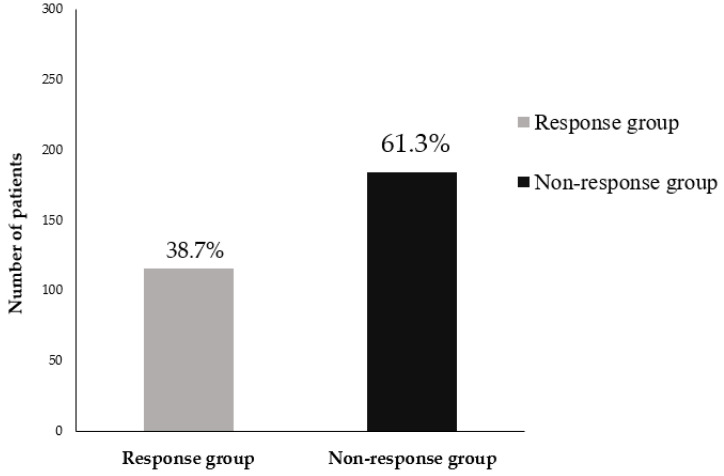
Incidence rate of bisphosphonate treatment failure in postmenopausal patients with osteoporotic vertebral fractures.

The incidence rate of bisphosphonate treatment failure was 61.3% (184/300 patients), which is relatively high compared to reports from other studies of patients with postmenopausal osteoporosis (Figure 3).

The incidence rate of 61.3% for bisphosphonate treatment failure was relative higher in the patients with severe osteoporosis (especially, OVFs) than in the patients with postmenopausal osteoporosis.

### 3.2. Comparison of Patent Demographics

All demographic data, including age, duration after menopause onset, follow-up duration, BMI, type of bisphosphonate, social history, and medical history, are summarized in Table 1. The number of subsequent vertebra fractures in the non-response group was 28 (15.2%).

### 3.3. Comparison of Radiological Factors between the Two Groups

In the radiological analysis of the fracture, the main location of the vertebral fracture was the thoracolumbar spine (62.4% in the response group and 38% in the non-response group) and lumbar spine (38% in the response group and 37% in the non-response group). Multiple OVFs were relatively higher in the non-response group (15.2%) than in the response group (4.3%) with statistical differences (*p* = 0.002). Regarding the MRI classification, the endplate type was mainly distributed in both groups (71.6% in the response group and 70.4% in the non-response group), with no significant difference (*p* = 0.891). The presence of an IVC sign was recorded to be 33.6% in the response group and 46.2% in the non-response group, with significant differences (*p* = 0.042). Considering the BMD and FRAX data, the initial mean BMD values of the spine were −2.57 in the response group and −3.12 in the non-response group (*p* < 0.001), while the last follow-up mean BMD values of the spine were −1.84 in the response group and −2.91 in the non-response group (*p* < 0.001). Overall, the differences in the mean BMD values of the spine were significantly different between the two groups (*p* < 0.001). The mean BMD values of the femur in the response group showed slight improvement from −2.08 to −1.86, but that of the non-response group worsened from −2.72 to −2.77, with statistical significance (all Ps < 0.001). The initial FRAX values of the major were 11.3% in the response group and 15.8% in the non-response group, respectively (*p* < 0.001). The initial FRAX values of the hip were 4.0% in the response group and 7.4% in the non-response group, with statistical differences (*p* < 0.001) (Table 2).

### 3.4. Comparison of Morphological Patterns between the Two Groups

In the morphological analysis for OVFs, the VBCR had decreased from 74.2% to 66.2% in the response group and from 73.0% to 62.2% in the non-response group. The 2-year follow-up PAHC showed 70.5% in the response group and 61.6% in the non-response group, with statistical significance (*p* = 0.003). The initial PMHC showed 77% in the response group and 71.7% in the non-response group, with statistical differences (*p* = 0.004). The 2-year follow-up PMHC showed 72.2% in the response group and 60% in the non-response group, with statistical significance (*p* < 0.001). Both groups did not present a difference of greater than 10° in the KA. (Table 3).

### 3.5. Logistic Regression Analysis for Bisphosphonate Treatment Failure

Univariate logistic regression analysis showed that age (*p* = 0.101), duration after menopause onset (*p* = 0.077), initial BMD of the spine (*p* < 0.001), and FRAX hip (*p* = 0.031) were associated with an increased risk of bisphosphonate treatment failure. Multivariate logistic regression analysis showed that the initial BMD of the spine and FRAX hip was associated with the risk of bisphosphonate treatment failure as indicated by an odds ratio of 1.962 and 1.320, respectively (all Ps < 0.001) (Table 4).

**Figure 4:**
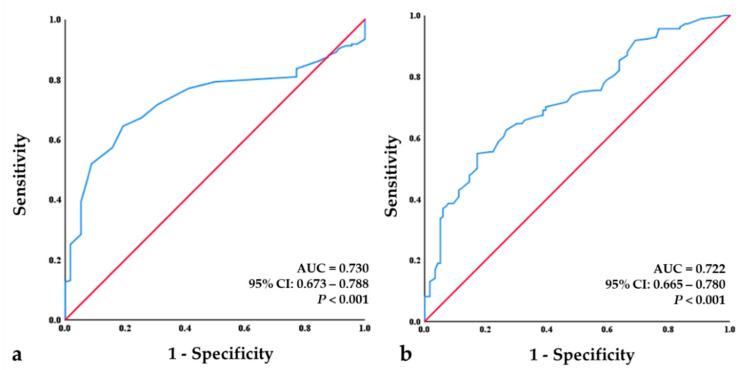
Receiver-operating characteristic (ROC) curve analysis for the initial BMD of the spine and initial FRAX hip. (**a**) ROC analysis revealed a cut-off value of −2.75 for the initial BMD of the spine as a good predictor (*p* < 0.001; area under the curve (AUC) = 0.730; sensitivity = 71.6%, specificity = 69.3%). (**b**) ROC analysis revealed a cut-off value −4.45% for the initial FRAX hip as a good predictor (*p* < 0.001; AUC = 0.722; sensitivity = 65.8%, specificity = 67.2%).

ROC curve analysis indicated an initial BMD value of the spine of −2.75 to be the optimal cut-off value, leading to a sensitivity value of 71.6% and specificity value of 69.3%. The area under the curve (AUC) for the initial BMD value of the spine was 0.730, which indicates a good predictive ability (*p* < 0.001). The ROC curve analysis also showed that an initial FRAX hip value of 4.45% to be the optimal cut-off value with a sensitivity 65.8% and specificity of 67.2%. The AUC for the FRAX hip value was 0.722, which indicates a good predictive ability (*p* < 0.001) (Figure 4).

## 4. Discussion

Globally, OVFs are one of the most common osteoporotic fractures and occur in 30% to 50% of people over the age of 60 years [1]. Although the mainstay of treatment for OVFs is a conservative approach using orthosis, OVFs still lead to high morbidity and mortality rates [30,31]. Over several decades, various anti-osteoporotic medications have been developed for preventing osteoporotic fractures and improving the bone density [7,18]. Anti-resorptive agents have been introduced with good efficacy and safety to prevent further fractures in patients with OVFs, but the use of bisphosphonate is negatively influenced by their low fracture healing potential [6,32]. However, despite the various anti-osteoporotic medications available, there is a lack of studies on choosing agents based on individual characteristics from radiological examinations [11,17]. Although the recent guidelines for osteoporosis recommend the use of anabolic agents in the severe osteoporosis group, clinicians face a practical issue where they have no choice but to use only bisphosphonate with the reason of the high costs and the difficulty in self-injecting anabolic agents daily [19,20,21]. Till date, no study has been reported in the literature on whether the choice of bisphosphonate can be justified.

Bisphosphonate is the most commonly prescribed agent for osteoporosis and is considered a first-line treatment option, with significant evidence supporting its abilities to increase bone density and reduce the fracture risk [18]. However, there have been many reports of patients with decreased bone loss who are diagnosed with fractures in spite of bisphosphonate treatment and who could hence be considered a bisphosphonate treatment failure [17]. Previous studies have reported various incidence rates (up to 53%) for inadequate effects of bisphosphonate on osteoporosis [33,34]. Díez-Pérez et al. documented an incidence rate of 42.5% for bisphosphonate treatment failure in the patients with postmenopausal osteoporosis from a multi-center, cross-sectional study [33]. However, differences in the incidence rates may be driven by patient-related factors including poor compliances with the oral administration of bisphosphonate and low calcium/vitamin levels. Caroli et al. reported an incidence rate of 25.8% when excluding patient-related factors [11]. In our study of postmenopausal women with OVFs, we reported an incidence rate of 61.3% for inadequate response to treatment with bisphosphonate in cases of severe osteoporosis, which is relatively high compared to the reports from other studies investigating patients with postmenopausal osteoporosis.

For the radiological analysis of BMD values, the differences in the BMD values also showed statistical differences for the spine and femur. Even the BMD values of the femur significantly deteriorated in the non-response group. Black et al. previously suggested that the reduction in the fracture risk using alendronate and zoledronic acid in postmenopausal women was contingent on the level of the BMD at baseline, similar to our results [4,5]. Thus, the non-responding group would show a greater decrease in BMD over time than the responding group.

In order to consider the proper criteria of bisphosphonate selection on the basis of the radiological factors, the logistic regression analysis showed that the BMD of the spine (odds ratio = 1.962) and FRAX hip (odds ratio = 1.32) at the point of diagnosis of OVF were risk factors for bisphosphonate treatment failure. In the ROC analysis, we found that the cut-off value for the BMD of the spine was −2.75 (sensitivity of 71.6% and specificity of 69.3%), with a relatively good predictive ability (AUC > 0.7; *p* < 0.001). Moreover, the cut-off value for the FRAX hip was −4.45 (sensitivity of 65.8% and specificity of 67.2%), with a relatively good predictive ability (AUC > 0.7; *p* < 0.001). Considering all patients in whom the OVFs were not limited with bisphosphonate medications, Díez-Pérez et al. suggested a low level of vitamin D to be a main risk factor [33]. Excluding factors related with poor compliance, Caroli et al. reported that a current smoking and a high level of bone turnover are important risk factors for an inadequate response to bisphosphonate treatment [11]. However, our results suggested that the initial BMD of the spine and the initial FRAX hip at the diagnosis of OVF are the most important risk factors for bisphosphonate treatment failure (all *p*-values < 0.001). Therefore, given our results, we suggest the choice of bisphosphonates for severe osteoporosis treatment in the case of OVFs should be made based on the values of the BMD of the spine and FRAX hip.

Although various randomized clinical trials have supported the preventive effect in osteoporosis-related skeletal events, cases with OVFs have been reported to exhibit aggregative morphological changes, including kyphosis and progressive collapse [35,36]. The presence of an IVC sign during the fracture-healing phase is considered an impaired healing signal following OVF that is associated with the risk of vertebra body instability [29]. The pathophysiology of IVC is avascular necrosis or nonunion of the vertebral body, which is a leading cause of progressive collapse or a possible delayed neurological compromise following OVFs [37,38]. There are some reports of factors related to the IVC sign, which included fracture location (especially thoracolumbar junction), MRI type (especially the mid-portion type), and fracture morphology [29,32,38]. As interesting results for morphological factors in this study, follow-up PAHC, and initial and follow-up PMHC were significantly lower in the non-response group than in the response group. These results mean that the anterior and middle regions of the vertebra body compared to the posterior region and adjacent segments were more compressed in the patients with OVFs who showed bisphosphonate treatment failure, which was also associated with the risk of vertebra body instability. Consistent with this result, the presences of IVC signs also showed significant differences between the two groups. Thus, the morphological factors regarding the middle region of the vertebral body are affected in bisphosphonate treatment failure, which is a possible risk factor of vertebral body instability.

Considering the mechanism of agents that have an anti-osteoporotic effect, bisphosphonate may interfere with the healing process of fractures. Several studies have documented no definite negative effects on fracture healing after bisphosphonate medication [39,40,41]. However, others have reported some results indicating delayed fracture healing with evidence of both the IVC sign and instabilities [32,38]. Our study showed all severe fracture morphology patterns for the patients with lower BMDs. Furthermore, the instability-related variables such as the PAHC and PMHC were significantly lower in patients with bisphosphonate treatment failure [29,37]. Thus, the response to bisphosphonate also indirectly affected the fracture-healing process for vertebral stability.

Our study has some limitations. First, the study design was a retrospective analysis, so we cannot discuss clinical outcomes regarding health-related quality of life, and our study may hold the possibility of selection bias from electronic medical records. The physical activity was not considered in this study due to the retrospective design. Compliances also were not accurately evaluated through a questionnaire, which is one of the limitations. Significant differences in the follow-up duration between the two groups were observed as another limitation of this study. Therefore, a randomized controlled trial considering the patient-driven factors will be necessary in future. Second, our study was performed at a single institute with a relatively small sample population. A real-world study may need to strengthen our results. Furthermore, choosing bisphosphonate for OVF is uncommon in the treatment of osteoporosis because of the potential impaired fracture-healing effect of bisphosphonate [38]. In patients with OVF, anabolic agents such as parathyroid hormones are preferred, despite the poor compliance owing to the difficulty in administering subcutaneous injections for an older age group [7,18]. Lastly, biomarkers regarding bone turnover were not included in our study. However, the main point of this study was to elucidate the risk factors for bisphosphonate treatment failure in patients with OVFs from a radiological point of view; thus, these factors were not included. A high turnover rate has also been reported as an important risk factor for an inadequate response to bisphosphonate in patients with osteoporosis [11]. Therefore, further studies will be needed at the molecular and cellular levels regarding bone turnover according to the inadequate response to anti-resorptive agents. Even so, our study suggests a good approach for selecting the anti-osteoporosis medications.

## 5. Conclusions

The bisphosphonate non-responder group showed a greater decrease in the BMD over time than the responder group. The initial BMD value of the spine and FRAX hip could be considered as radiological factors influencing bisphosphonate non-response in postmenopausal patients with OVFs. The failure of bisphosphonate treatment for osteoporosis has possible negative effects on the fracture healing process in OVFs.

## Figures and Tables

**Table 1:** Demographic data between the two groups.

Variable	Response Group (*n* = 116)	Non-Response Group (*n* = 184)	*p*
Age (years)	70.8 ± 6.7 *	72.1 ± 7.8 *	0.148
Duration after menopause onset (years)	20.0 ± 6.9 *	21.5 ± 7.8 *	0.108
Follow-up duration	5.3 ± 1.3 *	1.7 ± 1.1 *	<0.001
BMI (kg/m^2^)	23.6 ± 3.0 *	22.6 ± 3.4 *	0.232
Type of bisphosphonate (*n*)			0.587 ^†^
Alendronate	36 (31.0%)	65 (35.3%)	
Zoledronate	80 (69.0%)	119 (64.7%)	
Social history (*n*)			
Current smoker	10 (9.4%)	23 (12.5%)	0.392 ^†^
Current alcoholics	17 (14.7%)	30 (16.3%)	0.826 ^†^
Steroid medication	28 (24.1%)	38 (20.7%)	0.478 ^†^
Medical history (*n*)			
Hypertension	50 (43.1%)	60 (32.6%)	0.066 ^†^
CAOD	12 (10.3%)	17 (9.2%)	0.752 ^†^
CVA	15 (12.9%)	34 (18.5%)	0.206 ^†^
Diabetes mellitus	20 (17.2%)	32 (17.4%)	0.973 ^†^
Thyroid diseases	15 (12.9%)	24 (13.0%)	0.978 ^†^
Asthma/COPD	6 (5.2%)	12 (6.5%)	0.632 ^†^
Chronic kidney diseases	6 (5.2%)	3 (1.6%)	0.080 ^†^
Liver diseases	15 (13.0%)	20 (10.9%)	0.588 ^†^
Parkinson disease	4 (3.4%)	6 (3.3%)	0.930 ^†^
Dementia	16 (13.8%)	24 (13.0%)	0.852 ^†^
Rheumatic diseases	10 (8.6%)	13 (7.1%)	0.622 ^†^
Psychiatric diseases	17 (14.7%)	34 (18.5%)	0.391 ^†^

* All values are expressed as mean (± standard deviation). *p* values were calculated using the independent *t*-test for parametric data. ^†^ *p* values were calculated using the chi-square test. *n* = number; BMI = body mass index; CAOD = coronary artery occlusive disease; CVA = cerebrovascular accident; COPD = chronic obstructive pulmonary disease.

**Table 2:** Comparison of radiological factors between the two groups.

Variable	Response Group (*n* = 116)	Non-Response Group (*n* = 184)	*p*
Fracture			
Fracture location (*n*)			0.002 ^†^
Thoracic	5 (4.3%)	18 (9.8%)	
Thoracolumbar	62 (53.4%)	70 (38.0%)	
Lumbar	44 (38.0%)	68 (37.0%)	
Multiple	5 (4.3%)	28 (15.2%)	
MRI classification (*n*)			0.891 ^†^
Endplate type	83 (71.6%)	129 (70.1%)	
Mid-portion type	33 (28.4%)	55 (29.9%)	
Presence of an IVC sign	39 (33.6%)	85 (46.2%)	0.042 ^†^
BMD and FRAX			
BMD (T-score)			
Spine, initial	−2.57 ± 0.56 *	−3.12 ± 0.94 *	<0.001
Spine, last follow-up	−1.84 ± 0.56 *	−2.91 ± 0.88 *	<0.001
Spine, difference	0.72 ± 0.48 *	0.19 ± 0.58 *	<0.001
Femur, initial	−2.08 ± 0.61 *	−2.72 ± 0.76 *	<0.001
Femur, last follow-up	−1.86 ± 0.54 *	−2.77 ± 0.80 *	<0.001
Femur, difference	0.21 ± 0.38 *	−0.05 ± 0.50 *	<0.001
FRAX (%)			
Major	11.3 ± 4.1 *	15.8 ± 8.4 *	<0.001
Hip	4.0 ± 3.0 *	7.4 ± 5.7 *	<0.001

* All values are expressed as mean (±standard deviation). *p* values were calculated using the independent *t*-test. ^†^ *p* values were calculated using the chi-square test. *n* = number; MRI = magnetic resonance imaging; IVC = intravertebral vacuum cleft; BMD = bone mineral density; FRAX = fracture risk assessment tool.

**Table 3:** Comparison of morphological patterns between the two groups.

Variable	Response Group (*n* = 116)	Non-Response Group (*n* = 184)	*p*
VBCR (%)			
VBCR, initial	74.2 ± 14.2 *	73.0 ± 24.5 *	0.618
VBCR, 2-year follow-up	66.2 ± 24.2 *	62.2 ± 24.4 *	0.167
VBCR, difference	−8.4 ± 22.6 *	−10.5 ± 26.1 *	0.482
PAHC (%)			
PAHC, initial	79.1 ± 15.2 *	75.1 ± 23.9 *	0.110
PAHC, 2-year follow-up	70.5 ± 25.4 *	61.6 ± 24.2 *	0.003
PAHC, difference	−9.2 ± 26.2 *	−13.3 ± 29.3 *	0.214
PMHC (%)			
PMHC, initial	77.0 ± 17.6 *	71.7 ± 23.6 *	0.040
PMHC, 2-year follow-up	72.2 ± 25.5 *	60.0 ± 25.6 *	<0.001
PMHC, difference	−5.0 ± 26.0 *	−12.2 ± 32.4 *	0.052
KA (°)			
KA, initial	12.4 ± 7.0 *	13.3 ± 7.6 *	0.283
KA, 2-year follow-up	13.0 ± 8.2 *	14.6 ± 7.8 *	0.110
KA, difference	0.6 ± 9.5 *	1.0 ± 9.4 *	0.776
Cobb angle (°)			
Cobb angle, initial	14.6 ± 9.9 *	16.1 ± 12.8 *	0.252
Cobb angle, 2-year follow-up	18.7 ± 12.5 *	19.7 ± 12.5 *	0.474
Cobb angle, difference	4.1 ± 13.4 *	3.5 ± 14.9 *	0.733

*p* < 0.05 is significant. * All values are expressed as mean (± standard deviation). *p*-values were calculated using the independent *t*-test for parametric data and using the Mann–Whitney U test for non-parametric data. VBCR = vertebral body compression ratio; PAHC = percentage of anterior height compression; PMHC = percentage of middle height compression; KA = kyphotic angle.

**Table 4:** Univariate and multivariate regression analyses for bisphosphonate treatment failure.

Variables	Univariate Analysis (*n* = 300)			Multivariate Analysis (*n* = 300)		
	Beta	OR [95% CI]	*p*	Beta	OR [95% CI]	*p*
Age	−0.333	0.717 [0.481–1.067]	0.101	−0.312	0.732 [0.494–1.085]	0.120
Duration after menopause onset	0.356	1.427 [0.962–2.117]	0.077	0.340	1.404 [0.950–2.075]	0.088
BMD, spine initial	0.653	1.922 [1.346–2.744]	<0.001	0.674	1.962 [1.389–2.770]	<0.001
FRAX, hip	0.213	1.237 [1.020–1.500]	0.031	0.277	1.320 [1.184–1.471]	<0.001
Age	−0.333	0.717 [0.481–1.067]	0.101	−0.312	0.732 [0.494–1.085]	0.120

*p* < 0.05 is significant. *n* = number; OR = odd ratio; BMD = bone mineral density; FRAX = fracture risk assessment tool.

## Data Availability

Data collected for this study, including individual patient data, will not be made available.
